# A new hybrid metaheuristic method based on biogeography-based optimization and particle swarm optimization algorithm to estimate money demand in Iran

**DOI:** 10.1016/j.mex.2021.101226

**Published:** 2021-01-13

**Authors:** Sayyed Abdolmajid Jalaee, Alireza Shakibaei, Hamid Reza Horry, Hossein Akbarifard, Amin GhasemiNejad, Fateme Nazari Robati, Naeeme Amani Zarin

**Affiliations:** Department of Economics, Faculty of Management and Economics, Shahid Bahonar University of Kerman, Kerman, Iran

**Keywords:** Metaheuristic method, Money demand, Monetary and fiscal policies, Optimization algorithm

## Abstract

Money demand is one of the most important economic variables which are a critical component in appointing and choosing appropriate monetary policy, because it determines the transmission of policy-driven change in monetary aggregates to the real sector. In this paper, the data of economic indicators in Iran are presented for estimating the money demand using biogeography-based optimization (BBO) algorithm, particle swarm optimization (PSO) algorithm, and a new hybrid metaheuristic method based on biogeography-based optimization and particle swarm optimization algorithm (BBPSO). The data are used in two forms (i.e. linear and exponential) to estimate money demand values based on true liquidity, Consumer price index, GDP, lending interest rate, Inflation, and official exchange rate. The available data are partly used for finding optimal or near-optimal values of weighting parameters (1974–2013) and partly for testing the models (2014–2018). The performance of methods is evaluated using mean squared error (MSE), root mean squared error (RMSE), and mean absolute error (MAE). According to the simulation results, the proposed method (i.e. BBPSO) outperformed the other models. The findings proved that the recommended method was an appropriate tool for effective money demand prediction in Iran. These data were the result of a comprehensive look at the most influential factors for money market demand. With this method, the demand side of this market was clearly defined. Along with other markets, the consequences of economic policy could be analyzed and predicted.

• The article provides a method for observing the effect of economic scenarios on the money market and the analysis obtained by this proposed method allows experts, public sector economics, and monetary economist to see a clearer explanation of the country's liquidity plan.

• The method presented in this article can be beneficial for the policy makers and monetary authorities during their decision-making process.

## Graphical abstract

**Figure:**
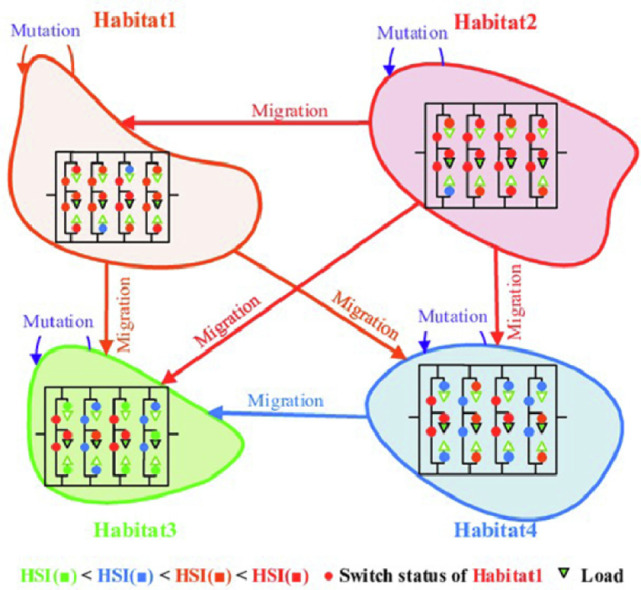

Specifications Table

**Table:** 

Subject Area:	Economics and Finance
More specific subject area:	Optimization methods, Monetary policy
Method name:	Biogeography-based optimization (BBO) algorithm Particle swarm optimization (PSO) algorithm BBPSO (hybrid BBO with PSO)
Name and reference of original method:	[1] MacArthur, R. and Wilson, E. (1967). “The Theory of Biogeography,” Princeton University Press, Princeton, NJ, USA. [2] Simon, D. (2008). “Biogeography-based optimization.” IEEE Transactions on Evolutionary Computation, vol. 12, no.6, pp. 702–713. [3] Kennedy, J. & Eberhart, R. (1995). "Particle Swarm Optimization." Proceedings of IEEE International Conference on Neural Networks. IV. pp. 1942–1948. doi:10.1109/ICNN.1995.488968.
Resource availability:	Time series data of Central Bank of Iran

## Introduction

Economics is the science of decision optimization. Therefore, prediction plays a major and effective role in optimizing, designing, and implementing monetary and fiscal policies. Since policymakers formulate and implement their strategies not only based on the current situation, but also based on the short- and long-term forecasts of key economic variables, the accuracy rate of predictions is a key to the success of these policies. In fact, achieving rapid and continuous growth requires adequate and sufficient information about the conditions of future economic variables. Moreover, predicting the future economic conditions and variables of a country is one of the main strategies in achieving appropriate information. The importance of this point has resulted in accelerating research in prediction and simulation models and techniques in recent decades. Adopting monetary and fiscal policies in the economy of any country and the maximum impact of these policies depends on perceiving the money demand function of that country [4].

Furthermore, recognizing the stability and forecast of money demand can be effective in controlling money supply in order to achieve economic goals and minimize the damage caused by the improper performance of the monetary system. The findings in advanced macroeconomics indicate that even when the growth rate of money is stable, hyperinflation is possible to occur in the economy. The cause of this potential hyperinflation can be the money demands of the private sector and the speed of adjusting the expectations. Therefore, due to the importance of money demand, using advanced techniques in predicting this fundamental variable can pave the way for monetary policymakers. The use of non-classical methods in identifying models and predicting the behavior of complex systems has long been common in scientific and even professional gatherings. In many complex systems, especially the non-linear ones, which make modeling and, then, predicting and controlling them through classical and analytical methods very difficult and sometimes even impossible, non-classical methods are used. These non-classical methods have features such as knowledge-based intelligence. Among these, we can mention cuckoo algorithms, firefly algorithm, particle swarm optimization, and biogeography, which are some of the most efficient forecasting methods in this field. Therefore, in this paper, by presenting a new hybrid method using biogeography algorithm and particle swarm algorithm, we will study the money demand function and the variables affecting it in Iran. The most accurate method will be provided to predict the money demand in Iran.

The paper is organized as follows: first, a brief introduction is presented. In the second section, the related works are discussed. Specifications of the BBPSO (hybrid biogeography-based optimization and particle swarm optimization algorithm) are presented in the third section. Section four contains optimization and method validation. In the fifth section, the model estimations are done and, finally, in the last section, the conclusion is stated.

## Related works

A few studies have proposed several models with different techniques. Different optimization techniques, e.g. birds, bats, and fireflies, are appropriate for forecasting these models. Ceylan et al. [5] developed harmony search (HS) technique to forecast Turkish transport energy demand in three forms (linear, exponential, and quadratic) and based on three factors including population, gross domestic product (GDP), and vehicle kilometers. The oil industry is a fundamental factor in the economy of Iran and the government's annual budget. It affects foreign trade, national capital, and developments in non-petroleum exports [6]. The energy consumption in Turkey was also determined by using the ant colony (ACO) by Toksari [7] with independent variables such as population, GDP, import, and export. Oil, natural gas, electric power, solar, wood, animal, and plant waste could be recognized as Iran's basic energy resource [8]. Kıran et al. [9] applied artificial bee colony (ABC) and particle swarm optimization (PSO) techniques to forecast electricity energy demand in Turkey in two forms (linear and quadratic) by using the selected socioeconomic and demographic variables that included GDP, population, import, and export

In [10], the authors proposed the bat algorithm (BA) for reducing the energy consumption in a wireless sensor network. This algorithm aims to select the best monitoring sensor node in the path toward decreasing energy consumption. Authors in [11] optimized the resource allocation (RA) problem in the Internet of things (IoT) based on the heuristic algorithm; this algorithm aims to achieve the goal of optimizing the RA and decreasing the total communication cost between resources and gate-ways. In [12], a new meta-heuristic algorithm was designed to solve the flexible dynamic job-shop problem with parallel machines. Sangaiah et al. [13] used the biogeography-based optimization (BBO) to reduce the parameters, and their experimental results illustrated the efficiency and feasibility of the proposed algorithm. Authors in [14] addressed a robust mixed integer linear programming model for LNG sales planning over a given time horizon and aimed to minimize the costs of the vendor.

## Methods and material

### BBO algorithm

This population-based evolutionary algorithm is inspired by the migration of animals and birds between islands. Biogeography is the study of the geographical distribution of biological species. Islands that are an appropriate place for geographical species to live have a high habitat suitability index (HSI). Features that determine HSI include rainfall, plant diversity, mapping features, soil, and temperature. High-HSI islands have many species that migrate to nearby islands.

High-HSI islands having low rates of immigration, as they have previously been populated by other species and cannot accept new ones. Lower HSI islands have a high rate of immigration due to their small population. Immigration of the new species to islands with low HSI may increase the area's HSI because the suitability of a location is commensurate with its geographical diversity. Using biographical geography for optimization has been the first step in using a natural process to solve optimization problems. Just as for other evolutionary algorithms such as GA, there are always operators such as mutation and crossover operator, in the BBO algorithm, migration and mutation operators cause the desired changes in the production process [1].

#### Immigration operator

Suppose we have a problem and a set of candidate solutions that are represented by the vector of correct numbers. Each correct number in the solution vector can be considered as one SIV (such as gene in GA). In addition, suppose that there are methods to determine the desirability of solutions. Optimal solutions include high HSI (habitat with many species) and weak solutions have low HSI (habitat with a low number of species). HSI in BBO is similar to fitness in other optimal population-based optimization algorithms. Each habitat (solution) in BBO has an immigration rate (λ) and emigration rate (μ), which are used to share possible information between the solutions. They are calculated using the following equations:

(1) $$\;\lambda {\;}_{k}=I\left(1-\frac{k}{n}\right)$$

(2) $$\;\mu {\;}_{k}=E\;\left(\frac{k}{n}\right)$$

where *I* and *E*, respectively, are the highest values of immigration and emigration, which can be included in the solutions, ki indicates the number of species in the imth habitat, which is a value between 1 and *n*, and *n* is the number of population members (*n* for the best solution and 1 for the worst solution). Every solution will be corrected based on other solutions with certain probability. If a solution is chosen for correction, the immigration rate (λ) is used to determine whether any of the SIVs in the solution need to be corrected. If an SIV in the Si solution is chosen for the correction, by using the emigration rate (μ) of the other solutions, we decide which of the solutions should cause randomly selected SIV migration to Si solution.

#### Mutation

**Fig. 1:**
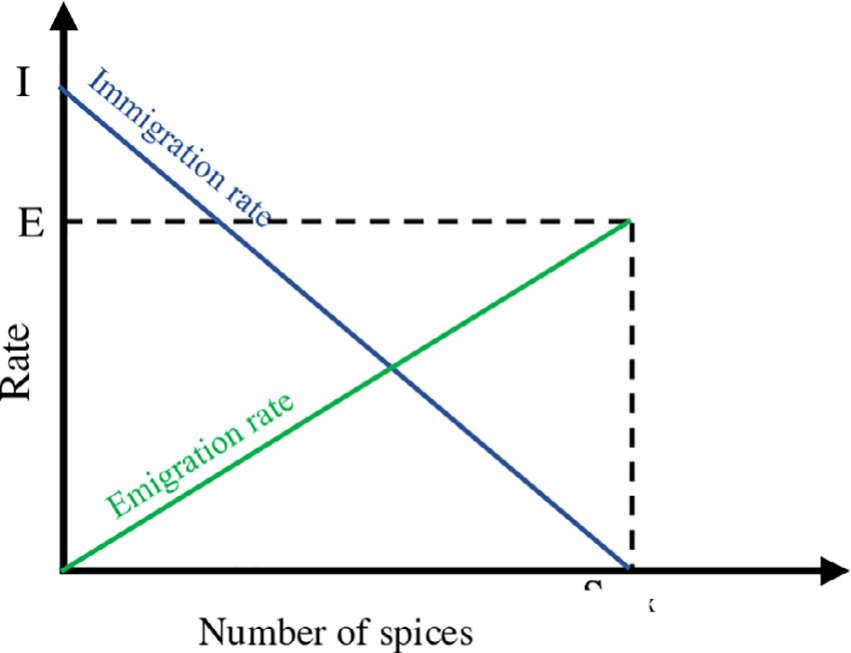
Migration rates vs. number of species.

Sudden changes can change the value of HSI in a habitat. They can also make the number of species differ from their equilibrium (unnatural substances brought to the region by water from neighboring habitats, disease, natural disasters, etc.). We model this as an SIV mutation in BBO and the probability of the number of species living in the habitat is used to determine the mutation rate.

(3) $$m_{s}=m_{max}\left(1-\frac{P_{s}}{P_{max}}\right)$$

where $m_{max}$ as maximum mutation rate is defined by the user and P_s_ is the probability, by which the habitat has precisely S species. Fig. 1 illustrates the model for immigration and emigration rates. This pattern of mutation leads to an increase in population diversity.

In general, the BBO algorithm is expressed as follows:

1- Initial value of the parameters: Defining the solution by SIV_s_ and habitats; determining the maximum number of species, highest fertility rates E and I, maximum value of mutation S_max_ value, $m_{max}$, and parameter of elitism [2];
2- Accidental production of primary solutions (habitats)
3. Using HSI values, number of species, S, type of immigration, λ, and rate of emigration to the outside as well as obtaining µ related to each habitat;
4. Correcting each non-elite habitat using immigration and emigration rates and re-calculating the migration operator and HIS;
5. Changing the probability of the number of species living in each habitat, mutating each non-elite habitat, and re-calculating the mutation operator and, then, the HSI value for each habitat;
6- Returning to Step 3 for the next iteration;
7- This circle can be completed after the predetermined number of generations or after finding an acceptable solution to the problem.

### Particle swarm optimization (PSO) algorithm

PSO is an optimization technique which works based on a population of initial responses. It is one of the most important and best algorithms introduced in the field of artificial intelligence. This technique was first designed and modeled based on the social behavior of a group of birds or fish by Eberhart and Kennedy [3].

In many cases, this method acts like evolutionary computational techniques, such as genetic algorithms. In this method, the system also starts with a population of initial responses and tries to find the optimal response by moving these responses in successive iterations. In this algorithm, each particle represents a response to a problem that moves randomly in the problem space. The displacement of each particle in the search space is influenced by itself and its neighbors, so the position of the other particles affects how the particle moves and searches. Each particle adjusts its location in the search space according to the best position it has ever been in and the best position in its entire neighborhood. The initial position of each particle is randomly determined in the search space by the uniform distribution within the problem definition range.

**Fig. 2:**
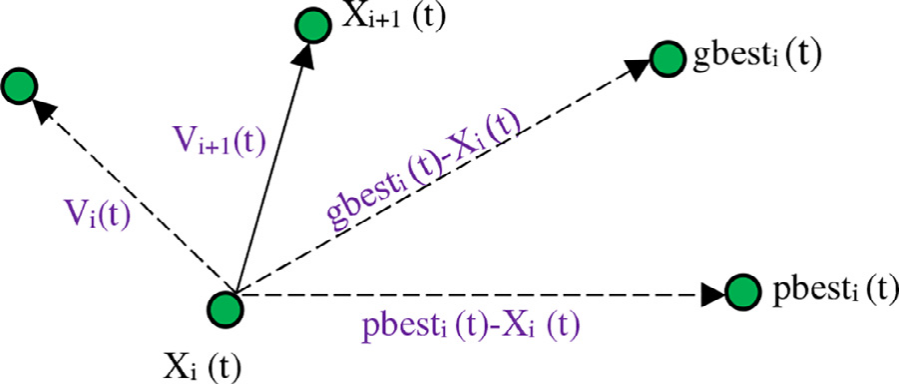
Movement of each particle.

Each particle is defined as the multidimensional (depending on the nature of the problem) with two values of $x_{i}^{d}\left(t\right){\;v}_{i}^{d}\left(t\right)$, which represent the spatial and velocity states of *d*th dimension of *i*th particle, respectively. Next times, the position of each particle is determined based on its own experience and that of its neighbors. If $x_{i}^{d}\left(t\right)$ is the next position d of the particle i at time t, the next position of the particle is obtained from the sum of the next position d of particle i at time t with the velocity of particle i. Particles are driven through a velocity vector. In the velocity vector, the result of social experience of neighboring particles and the individual experience of each particle are involved. Each particle updates its velocity by a linear combination of the individual part representing the use of personal knowledge and experience and the social part representing the experience of neighbors. In the individual part, the best position of the particle that has been achieved so far, pbest, is considered and, in the social part, the best position that all particles have achieved, gbest, is considered. Fig. 2 illustrates a schematic view of updating the position of a particle.

Each particle tries to change its position to achieve the best response using the following data and functions:

The current position $X_{\text{ij}}\left(t\right)$, current velocity $V_{\text{ij}}\left(t\right)$, distance between the current position and pbest, as well as distance between the current position and gbest. So, the velocity of each particle changes according to the following function:

(4) $$v_{ij}\left(t+1\right)=w.v_{ij}\left(t\right)+c_{1}.r_{1}\left(pbest_{ij}\left(t\right)-x_{ij}\left(t\right)\right)+c_{2}.r_{2}\left(gbest_{j}\left(t\right)-x_{ij}\left(t\right)\right)$$

where $v_{\text{ij}}\left(t\right)$ is j dimension of each particle in the t-iteration; $c_{1},c_{2}$ are positive constants that are used to weigh the individual and social parts and are called acceleration coefficients; $r_{1},r_{2}$ are random numbers between zero and one $(r_{1i}\left(t\right),r_{2i}\left(t\right)≃\;u\left(0,1\right)$ which maintain the random nature of the algorithm; and W is the inertia weight parameter [3].

The new position of each particle is obtained from the sum of the previous position and the new velocity, which is determined according to the following equation:

(5) $$x_{ij}\left(t+1\right)=x_{ij}\left(t\right)+v_{ij}\left(t+1\right)$$

### Proposed method (BBPSO)

Meta-heuristic algorithms have been widely considered by researchers due to the following advantages: ease of implementation and modeling a problem, high flexibility against changing parameters, finding approximate responses by using global and local search operators for complex problems, possibility of combining and improving by other computational methods, and having many solutions to avoid local optimal points. One of the widespread disadvantages of meta-heuristic algorithms is the premature convergence to the optimal local points of the problem. In this case, a function gets stuck in the local points and reports the local extremum point as the absolute extremum point. BBO is a population-based evolutionary algorithm dominated by mathematical biogeography. PSO is a simple and powerful algorithm in solving many optimization problems.

The weakness of the BBO algorithm is that it provides the optimal local solution in many cases. One way of increasing search power in the response space and improving the variety of responses is to combine the basic algorithm with other techniques. PSO algorithm is a simple and fast algorithm that can improve the weaknesses of the BBO algorithm. This algorithm can create more variety in the responses of BBO algorithm due to length (duration) of the step and fast movement in the response space; therefore, it seems to be an appropriate choice. In the proposed algorithm called BBPSO, a combination of these algorithms is used. In BBPSO, both algorithms use the same population and share the responses.

The algorithm starts with creating the initial population and, at each iteration, one of the two algorithms makes changes to the population. BBO tries to improve the responses by migration and mutation, and PSO does it by moving the particles. In the basic BBO algorithm, the best values obtained from the iteration of the algorithm replace the worst values in the selection process. This is a one-to-one process and the best response replaces the worst one. If the response is duplicated, a random response replaces the previous one. In BBPSO algorithm, the selection method is developed. If the response is duplicated, the next best response is replaced, provided that a defined separation criterion which ensures higher variety of responses is improved. In BBO algorithm, migration and mutation operators are based on habitat migration rates; but in PSO algorithm, the best values of the objective function of each particle group and the overall optimal value play a role in finding the next particle location.

In combining the two algorithms and because BBO algorithm is implemented in some iterations, it is necessary to store the historical data of each habitat to be used in PSO algorithm in order to create an integration. Alternative responses have no historical data and the value of the best place they are in is considered equal to their current place. The steps of the BBPSO algorithm are described below.

#### Determining the values of algorithm parameters

In BBO algorithm, parameters such as E, I, and migration rate function are defined as in the basic algorithm. E = I = 1 and migration rate function are linear. Mutation rate (Mmax) and maximum number of species (Smax) are other parameters of BBO algorithm. The values c1, c2, w, and Vmax must also be defined for the PSO algorithm. These values are also determined as the basic algorithm and *w* = 1, c1 = c2 = 2. In hybrid algorithm, a parameter called BorPC is defined, which is a value between 0 and 1, and indicates the probability of selecting BBO algorithm in each iteration. The closer this value is to 1, the closer the algorithm is to BBO algorithm; the closer it is to zero, the closer the algorithm is to PSO algorithm. In this article, this value is considered as 0.5 [15].

#### Determining the initial population

The initial population is a matrix with dimensions N*D, in which N is the number of habitats (clusters) and D is the number of decision variables. If X_i_ is response i, we have X_i_ = (X_i1_, X_i2_…, X_iD_). To create the initial response, random numbers are generated in the response space for each X_ij_ and the initial response space is formed. There is a range for each of the decision variables and we show it with (l_j_, u_j_), where u_j_ is the upper bound and l_j_ is the lower bound for variable i to produce random numbers:

(6) $$X_{ij}=L_{j}+rand\left(0,1\right)*\left(u_{j}-L_{j}\right)$$

#### Updating responses

A random number between 0 and 1 is selected and one of the two basic algorithms is selected according to the BorPC value.

##### BBO algorithm

The amount of objective function is calculated for each habitat. After saving the location of each habitat, sorting is done based on the value of the objective function. Migration rates are set and migration operator is applied. The values of the objective function are re-calculated and the probability of mutation of each habitat is determined. Mutation operator is applied. The values of the objective function are re-calculated and the values of pbest and gbest are updated for each response. The particle velocity in PSO algorithm is the difference between the new particle location and their previous location. Therefore, the particle velocity is set to the changes in each habitat and the algorithm ends.

##### *PSO algorithm*

Particles are sorted according to their initial positions because the historical data are saved based on their original positions. Also, the particle position may have been changed under BBO algorithm. The velocity of each particle and their next location is determined. The value of objective function is determined for each response, the pbest and gbest values are updated, and the algorithm ends.

### Elite selection

Updated responses and previous responses are sorted by the value of objective function. The best updated responses replace the worst previous responses. The number of substitutions is the initial parameters of the algorithm. If a response is repetitive, the best next response is replaced. If the number of non-repetitive responses is not equal to the number of defined elites, the random response replaces the previous one. On the other hand, the values of the alternative responses must be in the range of (l_j_, u_j_).

**Fig. 3:**
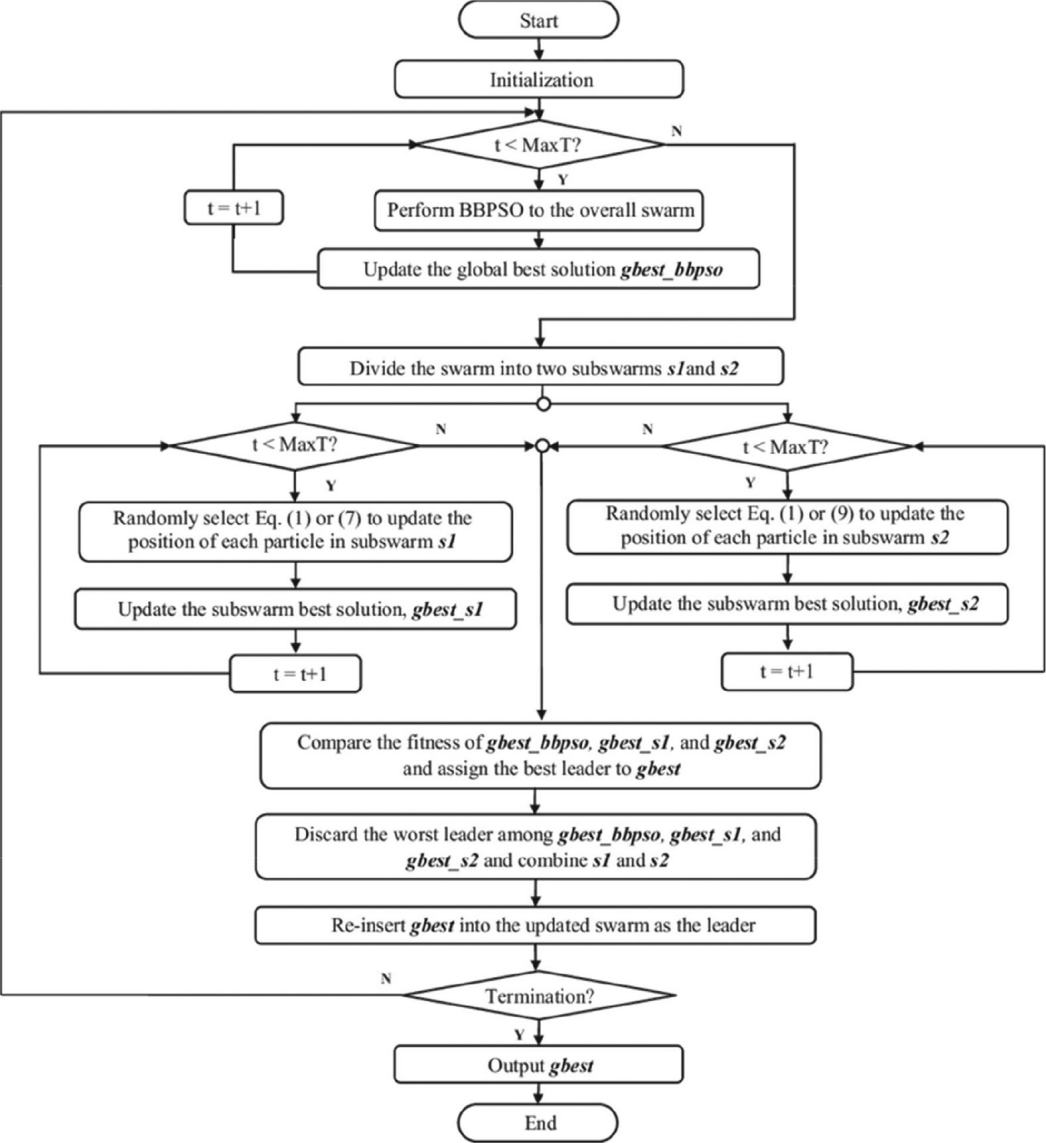
The flowchart of the proposed BBPSO algorithm.

Fig. 3 shows a flowchart of the proposed BBPSO algorithm.

## Optimization and method validation

The aim of this paper is to introduce a new hybrid method to estimate money demand in Iran. BBO, PSO, and BBPSO are developed to estimate money demand values based on the true liquidity, consumer price index, GDP, lending interest rate, inflation, consumer prices, and official exchange rate**.** For this purpose, the form of citizenship in accordance with Eq. (7) is used to analyze the methods [16].

(7) $$y=f\left(x_{1},x_{2},x_{3},x_{4}\right)$$

The models have been developed in two forms: linear and exponential.

The linear form of the equation to estimate money demand model is written as follows:

(8) $$Y_{\text{linear}}=\;C_{1}+C_{2}X_{1}+C_{3}X_{2}+C_{4}X_{3}+C_{5}X_{4}$$

The exponential form of the equation to estimate money demand model is written as follows:

(9) $$Y_{exponential}=C_{1}+C_{2}X_{1}^{c_{3}}+C_{4}X_{2}^{c_{5}}+C_{6}X_{3}^{c_{7}}+C_{8}X_{4}^{c_{9}}$$

where y represents the (true liquidity / consumer price index) and $X_{i}$ values represent the four independent variables used as the predictors of y ($X_{1}$ is GDP, $X_{2}$ is lending interest rate, $X_{3}$ is inflation, and $X_{4}$ is official exchange rate) [17,18].

The fitness function of the algorithm corresponds to Eq. (10).

(10) $$Min\;F\left(x\right)=\sum_{j=1}^{k}{\left(y_{actual}^{i}-y_{estimated}^{i}\right)}^{2}$$

In addition, error metrics MSE,^1^ MAE,^2^ and RMSE^3^ are used to compare the performance of the proposed method in the form of two linear and exponential models in order to provide the best method analysis. The formula for this error is specified in the form of Eqs. (11)-(13) [[19], [20]–21].

(11) $$MSE=\frac{1}{n}\sum_{i=1}^{45}{\left(y_{actual}^{i}-y_{estimated}^{i}\right)}^{2}\;for\;n=1,2,\ldots ,45$$

(12) $$MAE=\frac{1}{n}\sum_{i=1}^{45}\;\left|y_{actual}^{i}-y_{estimated}^{i}\right|\;forn=1,2,\ldots ,45$$

(13) $$\text{RMSE}=\sqrt{\frac{1}{n}\sum_{i=1}^{45}{\left(y_{actual}^{i}-y_{estimated}^{i}\right)}^{2}\;}forn=1,2,\ldots ,45$$

Money demand from 1974 to 2018 is considered as a case in point in this paper. The available data are partly used for finding the optimal, or near-optimal, values of the weighting parameters (1974–2013) and partly for validating the methods (2014–2018). [22]

The following steps are conducted for estimating money demand in Iran between 1974 and 2018:

**Step 1:** True liquidity, consumer price index, GDP, lending interest rate, inflation, consumer prices, and official exchange rate need normalization according to Eq. (14):

(14) $$X_{N}=\left(X_{R}-X_{min}\right)/\left(X_{max}-X_{min}\right)$$

X_N_: Normalized value, X_R_: Value to be normalized, X_min_: Minimum value in all the values for related variable, X_max_: Maximum value in all the values for the related variable. The X_min_ and X_max_ values for each variable are selected between 1974 and 2013 and are shown in Table 1.

**Table 1:** Values for normalization.

VARIABLE	X_min_	X_max_
True liquidity (Billion IIRR)	7507.246	139596.7
Consumer price index	0.069	109.6
GDP (Billion IRR)	1707	14,807,101
Lending interest rate (%)	6	22.2
Inflation, Consumer prices (Annual %)	6.9	49.4
Official exchange rate (IRR)	66.9	34,214

**Step 2:** The proposed methods are used in order to determine the corresponding weighting factors ($C_{i}$) for each model according to the lowest objective functions.

**Step 3:** The best results of Step 2 for each model and less average relative errors in the testing period are chosen (i.e. the related data from 2014 to 2018). The best-obtained weighting factors by BBO, PSO, and BBPSO methods for linear and exponential models are shown in Table 2.

**Table 2:** The best-obtained weighting factors by BBO, PSO, and BBPSO algorithms.

Methods	C1	C2	C3	C4	C5	C6	C7	C8	C9
$Y_{\mathrm{BBO}-\mathrm{MD}{*}_{\mathrm{linear}}}$	−0.117	0.874	−0.326	−0.477	0.379	–		–	–
$Y_{\mathrm{BBO}-MD_{\mathrm{exponential}}}$	−0.446	0.816	0.350	−0.335	0.025	−0.539	0.221	0.838	0.165
$Y_{\mathrm{PSO}-MD_{\mathrm{linear}}}$	−0.501	0.704	−0.506	−0.287	0.925	–	–	–	–
$Y_{\mathrm{PSO}-MD_{\mathrm{exponential}}}$	−0.502	0.789	0.227	−0.100	0.186	−0.307	0.826	0.455	1.002
$Y_{\mathrm{BBPSO}-MD_{\mathrm{linear}}}$	−0.100	0.710	−0.422	−0.563	0.708	–	–	–	–
$Y_{\mathrm{BBPSO}-MD_{\mathrm{exponential}}}$	−0.445	0.907	0.722	−0.344	0.105	−0.441	0.662	0.656	0.588

*money demand.

The obtained values of MSE, RMSE, and MAE for methods are presented in Table 3.

**Table 3:** Comparing performance evaluation of methods.

Performance criteria			
Algorithm models	MSE	MAE	RMSE
$Y_{\mathrm{BBO}-MD_{\mathrm{linear}}}$	0.105	0.262	0.324
$Y_{\mathrm{BBO}-MD_{\mathrm{exponential}}}$	0.015	0.086	0.123
$Y_{\mathrm{PSO}-MD_{\mathrm{linear}}}$	0.296	0.505	0.544
$Y_{\mathrm{PSO}-MD_{\mathrm{exponential}}}$	0.009	0.078	0.098
$Y_{\mathrm{BBPSO}-MD_{\mathrm{linear}}}$	0.104	0.255	0.323
$Y_{\mathrm{BBPSO}-MD_{\mathrm{exponential}}}$	0.006	0.062	0.081

It can be seen that there is a good agreement between the results obtained from BBO, PSO, and BBPSO methods, but the BBPSO_$MD_{\text{exponential}}$ model outperforms the others.

**Fig. 4:**
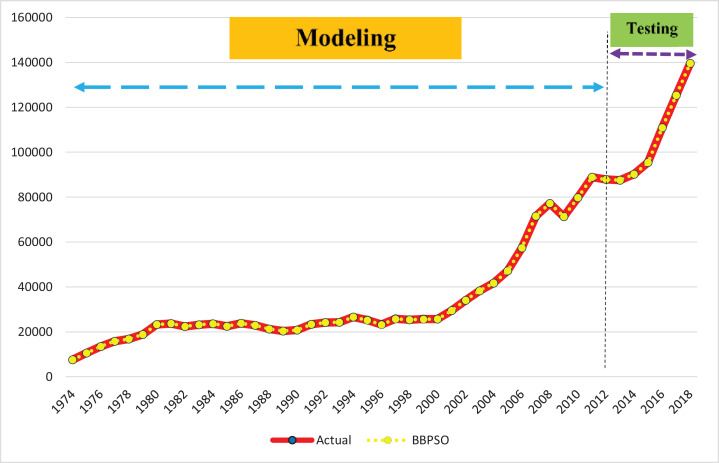
Comparing actual data and predicted values with BBPSO.

Table 4 and Fig. 4 show the performance of the BBPSO method for modeling and forecasting money demand function.

**Table 4:** Performance evaluation of BBPSO method in testing period (2014–2018).

years	2014	2015	2016	2017	2018	Average
Actual data	90184.2	95473.3	110918.7	125339.0	139596.7	–
$Y_{\mathrm{BBPSO}-MD_{\mathrm{exponential}}}$	90178.3	95369.3	110852.3	125326.3	139592.3	–
Relative error (%)	0.019	0.135	0.128	0.215	0.240	0.147

Table 5 indicates a comparison of the different models introduced in the introduction and present study.

**Table 5:** Comparing various models introduced in the introduction and present study ^a^.

Source	Method	Target-Country	Average relative errors (%)
Toksarı [8]	Ant colony algorithm	Total energy- Turkey	1.070
Ceylan et al. [5]	Harmony search	Total energy-Turkey	21.740
	Harmony search	Total energy-Turkey	13.410
	Harmony search	Total energy-Turkey	39.320
Assareh et al. [8]	Genetic algorithm	Oil-Iran	2.830
	Genetic algorithm	Oil-Iran	1.720
	Particle swarm optimization	Oil-Iran	1.400
	Particle swarm optimization	Oil-Iran	1.360
Behrang et al. [6]	Gravitational search algorithm	Oil-Iran	1.140
	Gravitational search algorithm	Oil-Iran	1.520
	Gravitational search algorithm	Oil-Iran	1.430
	Gravitational search algorithm	Oil-Iran	3.320
	Gravitational search algorithm	Oil-Iran	1.330
Kıran et al. [9]	Particle swarm optimization	Electricity-Turkey	3.990
	Particle swarm optimization	Electricity-Turkey	4.406
	Artificial bee colony	Electricity-Turkey	3.200
	Artificial bee colony	Electricity-Turkey	4.470
Present study	Hybrid biogeography-based optimization and particle swarm optimization algorithm (BBPSO)	Money demand-Iran	0.147

## Conclusion

Demand-side data on the money market clearly determine the uncertain segment of the money market. The money demand function is useful for policymakers and researchers in macroeconomics, public sector economics, and monetary economics. Not only can money demand function clearly define the demand side of this market, but also the consequences of economic policy can be analyzed and predicted along with other markets. The study aimed to show the authorities the significance of utilizing option estimating methods. In this paper, BBO, PSO, and a proposed method (BBPSO) based on the biogeography-based optimization (BBO) with particle swarm optimization were utilized appropriately to calculate money demand in Iran via investigating the true liquidity, consumer price index, GDP, lending interest rate, inflation, and official exchange rate. As presented in Table 5, the empirical results of the data in Iran exhibited the BBPSO method's accuracy was more precise than the other methods. The BBPSO's success in such a study suggested it may apply as a practical instrument for economic analysis in various areas with more theoretical specifications complexity. These data were the result of a comprehensive look at the most influential factors for money market demand. That is the effects of production showed interest rates, inflation, and exchange rates on people's behavior toward holding money. Therefore, it is possible to observe the effect of economic scenarios on the money market using BBPSO. This method allows policymakers to see a clearer explanation of the country's liquidity plan. Forecasting money demand function can as well be studied by neural networks or other new metaheuristics including harmony search, simulated annealing, etc. The results of different methods may be compared with those of BBPSO method.

## Declaration of Competing Interest

The authors declare that they have no known competing financial interests or personal relationships that could have appeared to influence the work reported in this paper.

## Footnotes

1 Mean squared error

2 Mean absolute error

3 Root mean squared error
